# High Neutrophil-to-Albumin Ratio Predicts Postoperative Pneumonia in Aneurysmal Subarachnoid Hemorrhage

**DOI:** 10.3389/fneur.2022.840858

**Published:** 2022-04-07

**Authors:** Xin Zhang, Sheng Zhang, Congkai Wang, Ran Liu, Aimin Li

**Affiliations:** ^1^Lianyungang Clinical College, Nanjing Medical University, Lianyungang, China; ^2^Qingpu Branch, Zhongshan Hospital Affiliated to Fudan University, Shanghai, China; ^3^The First Affiliated Hospital, Nanchang University, Nanchang, China

**Keywords:** aneurysmal subarachnoid hemorrhage, inflammation, pneumonia, neutrophil, albumin

## Abstract

**Background and Aim:**

There is still an absence of objective and easily accessible biomarkers despite the variety of risk factors associated with postoperative pneumonia (POP) in patients with aneurysmal subarachnoid hemorrhage (aSAH). In the present study, we have thus evaluated the relationship between the neutrophil-to-albumin ratio (NAR) and POP in patients with aSAH.

**Methods:**

Several consecutive patients (*n* = 395) who had undergone clipping or coiling of the aneurism were retrospectively assessed, of which we were able to analyze the existing population data and the related baseline variables. The patients were divided into POP and revealed not to occur. To identify independent predictors, we used the recipient operation feature (receiver operating characteristic, ROC) curve and a logic regression analysis.

**Results:**

This cohort was based on POP that occurred in 78 out of the 395 patients (19.7%), and these revealed a significantly higher NAR than those without (0.31 [0.25–0.39] vs. 0.23 [0.18–0.28]; *p* < 0.001**)**. On the other hand, a multivariate logistic regression analysis showed that NAR (odds ratio = 1.907; 95% confidence interval, 1.232–2.953; *p* = 0.004) was independently associated with a POP after due adjustment for confounders. Moreover, the predictive performances of NAR for POP were also significant (area under the ROC curve [95% CI] 0.775 [0.717–0.832]; *p* < 0.001).

**Conclusion:**

The elevation of NAR at admission in patients with aSAH might help predict POP.

## Introduction

Aneurysmal subarachnoid hemorrhage (aSAH) represents a devastating and life-threatening type of hemorrhagic stroke (1–3). Despite the advancements in the current treatment of intracranial aneurysms, including microsurgery and endovascular coil embolization, both disability and mortality rates of aSAH have remained high (4–6).

The aSAH in patients was also found to be accompanied by vasospasm and other non-neurologic complications, including postoperative pneumonia (POP), a central venous catheter (CVC)-associated infections, and urinary tract infection, among others (7), whereas studies have shown that from 13 to 37% of patients with aSAH may have POP impacted after surgical treatment (7–9).

The current research is thus aimed at finding early risk factors for POP, which might help identify high-risk patients for aggressive monitoring and therapeutic interventions.

Numerous risk factors of POP have been identified, most are based on clinical features, including age, aSAH severity, and ventilator use (7, 10, 11). Biomarkers of inflammation, such as the neutrophil-to-lymphocyte ratio (NLR), have recently gained particular interest during the last years (12) with plenty of evidence indicating that the neutrophil-to-albumin ratio (NAR) is an independent risk factor for cancer, sepsis, and in-stent restenosis undergoing both carotid angioplasty and stenting and ST-segment elevation myocardial infarction (STEMI) (13–16).

However, the mechanistic link between NAR and aSAH remains unstudied, which is why we sought to investigate in the present study the relationship between NAR and POP after aSAH, on the one hand, and whether NAR at an early stage may help to identify patients at high risk of POP, on the other (17).

## Materials and Methods

### Study Population

We reviewed the electronic medical records of 395 patients with aSAH who underwent microsurgery or endovascular coil embolization retrospectively, spanning a period ranging from December 2016 to December 2019 at the Lianyungang College of Nanjing Medical University. The individual patient's informed consent was not necessary for the elaboration of this study. Likewise, the Lianyungang First Hospital's Ethics Committee approved the study.

The criteria for inclusion were as follows: (1) age ≥ 18 years of all patients, who suffered their first SAH ever, admitted to our hospital within 24 h after onset; (2) SAH was confirmed by computer tomography (CT), whereas aneurysms were confirmed by computed tomographic angiography (CTA) or digital subtraction angiography (DSA); (3) laboratory investigations were obtained within 24 h after admission in the context of a first examination; and (4) surgical clipping or endovascular coiling of the aneurysm was performed. Additionally, the criteria for exclusion were listed as follows: (1) patients with other possible causes of SAH (e.g., craniocerebral trauma, arteriovenous malformation, hypertensive intracerebral hemorrhage, and brain tumors); (2) patients with a history of connective or autoimmune diseases, uremia, blood system diseases, malignant tumors, cirrhosis, chronic lung or heart diseases; (3) patients who had been admitted to the hospital with pneumonia; and (4) previous use of antibiotics, systemic glucocorticoids, immunosuppressive agents, or immunotherapy within 1 month before admission; and (5) patients who had undergone any surgery in the previous month or an acute inflammatory/infectious disease in the past 3 months.

### Data Collection

We collected general demographic data for patients, including age, sex, past medical history, radiological data, and laboratory results, and the severity was classified by the World Federation of Neurosurgical Societies (WFNS) grade. In contrast, the amount of bleeding was classified following the modified Fisher (mFisher) grade. Therefore, WFNS grade ≥ 3 and mFisher grade ≥ 3 upon admission were considered in this study as severe aSAH.

According to the modified Centers for Disease Control and Prevention (CDC) criteria (18), POP was defined as the set of lower respiratory tract infections that occur within 30 days after an operative procedure, which pertains to the following: (1) a probable POP could not be diagnosed based on the admission or the follow-up chest X-ray, and it could neither be explained by another diagnosis; (2) proven POP had a confirmed change in diagnostic on at least one image of the chest X-ray. Patients with probable/proven pneumonia were considered cases for this study by the modified CDC criteria (19, 20), whereas all patients with pneumonia before admission were excluded.

### Statistical Analysis

Although the statistical analysis was conducted using SPSS version 26.0 (IBM Corporation, Armonk, New York, USA) and MedCalc 9.6.4.0 (Mariakerke, Belgium), the GraphPad Prism 8.0 software (GraphPad Software, La, Jolla, CA, USA) was used for figure production. The continuous variables which were non-normally distributed were expressed as median (interquartile range) and were compared by using the Mann–Whitney *U-*test. In contrast, the categorical variables were presented as counts (percentages) and were compared by using the Pearson chi-square test or the Fisher exact test. All variables with a significant value of *p* < 0.05 obtained from the univariate analysis were entered into a binary multivariate logistic regression model, which was then used to determine independent predictors of POP.

We used the receiver operating characteristic (ROC) curve and the area under the ROC curve (AUC) to determine the predictive value of NAR for POP and optimal cut-off values. We also compared the predictive power between NAR and conventional markers (e.g., age, WFNS grade, and NLR) using the *Z*-test. Finally, we dichotomized the cohort to evaluate the basic characteristics of subjects with a high NAR, basing it on the optimal cut-off values of NAR and drawing a comparison between high and low NAR groups. A value of *p* < 0.05 was considered statistically significant.

## Results

Several patients with aSAH (*n* = 395) were considered for this retrospective analysis, out of which 78 (19.7%) patients had POP after operative treatment (Table 1). The group with POP was characterized by older age and severe clinical conditions upon admission (higher WFNS grade, mFisher grade, and intraventricular hemorrhage; Table 1). They also proved to have a higher rate of ventilator use and higher neutrophil, lymphocyte, D-dimer, and NLR on admission.

**Table 1:** Comparison of demographic and clinical data in patients with aneurysmal subarachnoid hemorrhage according to the occurrence of postoperative pneumonia.

**Characteristics**	**Non-POP group** **(*n* = 317)**	**POP group** **(*n* = 78)**	***P-*value**
**Demographics**			
Age(years)	61.0(51.0-65.0)	64.0(55.5-69.0)	0.001
Gender (female)	223(70.3%)	51(65.4%)	0.412
**Prior medical history**			
Hypertension	219(69.1%)	63(80.8%)	0.286
Diabetes	17(5.4%)	7(9.0%)	0.05
**Admission status**			
WFNS grade			<0.001
< Grade III	281(88.6%)	39(50.0%)	
≥Grade III	36(11.4%)	39(50.0%)	
**Admission CT**			
mFisher grade			<0.001
< Grade III	277(87.4%)	41(52.6%)	
≥Grade III	40(12.6%)	37(47.4%)	
IVH	60(18.9%)	39(50.0%)	<0.001
**Aneurysm characteristics**			
Location			0.755
anterior circulation	304(95.9%)	74(94.9%)	
posterior circulation	13(4.1%)	4(5.1%)	
Size of aneurysm (mm)			0.246
<5	166(52.4%)	33(42.3%)	
5-10	140(44.2%)	40(51.3%)	
11-25	10(3.2%)	5(6.4%)	
>25	1(0.3%)	0(0.0%)	
Treatment			0.733
Clip	273(86.1%)	66(84.6%)	
Coil	44(13.9%)	12(15.4%)	
Mechanical ventilator used	16(5.0%)	35(44.9%)	<0.001
**Lab values on admission**			
Neutrophil, ×10^9^/L	9.21(7.28-11.30)	12.24(10.16-15.26)	<0.001
Lymphocytes, ×10^9^/L	0.93(0.64-1.29)	0.84(0.55-1.15)	0.032
NAR	0.23(0.18-0.28)	0.31(0.25-0.39)	<0.001
NLR	9.86(6.16-15.07)	14.92(11.01-21.87)	<0.001
Platelet count, ×10^9^/L	206.00(170.50-245.50)	208.00(172.00-265.50)	0.506
D-Dimer, ng/ml	353.00(186.50-680.50)	635.50(264.25-1306.25)	<0.001
Plasma fibrinogen, ug/ml	2.93(2.56-3.32)	2.88(2.51-3.46)	0.705

**Figure 1:**
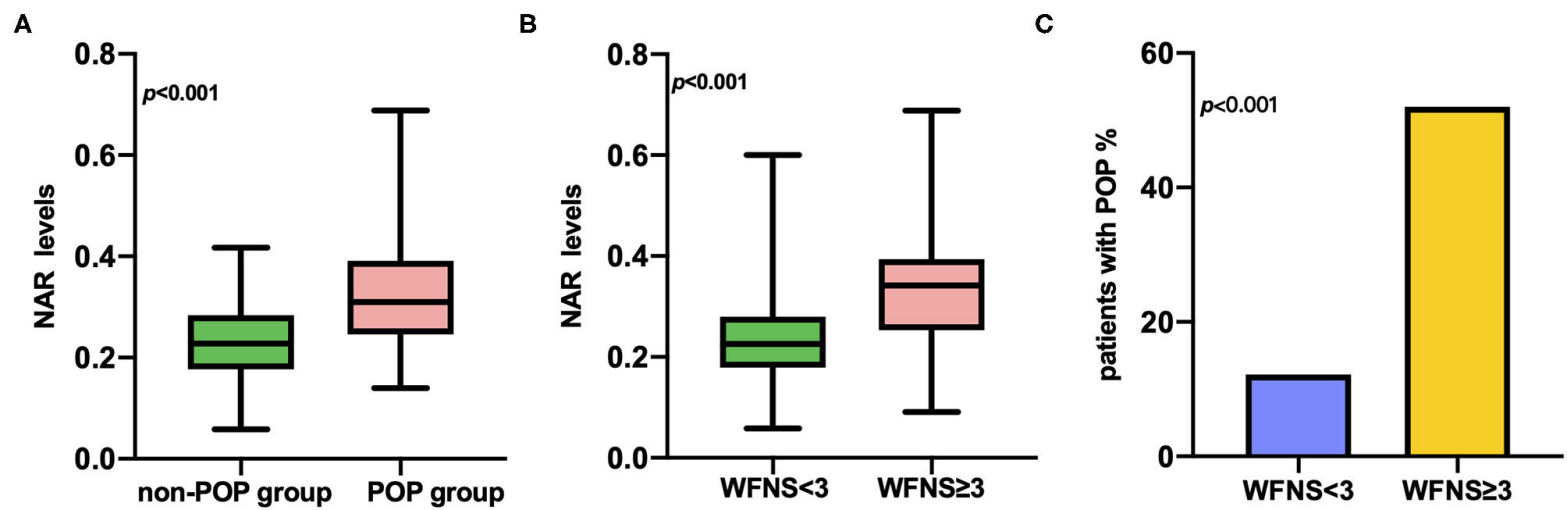
**(A)** Difference of NAR levels between POP group and non-POP group (0.31[0.25–0.39] vs 0.23[0.18–0.28]; *P* < 0.001). **(B)** Relationship between NAR levels and WFNS grade in patients with aneurysmal subarachnoid hemorrhage (WFNS <3 vs. WFNS ≥ 3, 0.23[0.22–0.24] vs. 0.34[0.31–0.37]; *P* < 0.001). **(C)** Relationship between rates of POP and WFNS grade in patients with aneurysmal subarachnoid hemorrhage (WFNS <3 vs. WFNS ≥ 3, 12.2% vs. 52.0%; *P* < 0.001).

The POP group showed a significantly higher NAR on admission than that of the non-POP group (0.31 [0.25–0.39] vs. 0.23 [0.18–0.28]; *p* < 0.001; Table 1; Figure 1A). In addition, patients with severe aSAH (WFNS ≥ 3) exhibited significantly higher NAR levels (0.23 [0.22–0.24] vs. 0.34 [0.31–0.37]; *p* < 0.001) and higher rates of POP than the rest (12.2 vs. 52.0%; *p* < 0.001) (Figures 1B,C).

**Figure 2:**
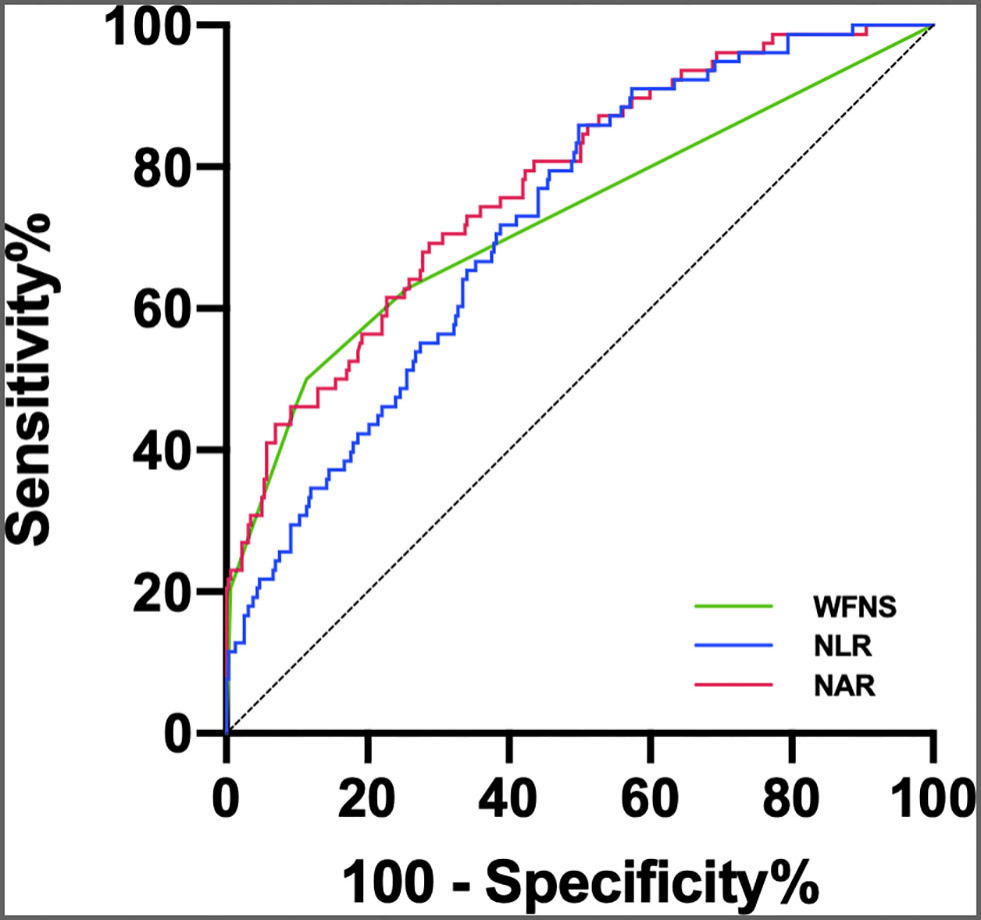
ROC curve analysis comparing WFNS grade, NLR and NAR at admission for identifying POP following aSAH. The AUCs of WFNS grade, NLR and NAR were 0.725 (95% 0.654–0.796), 0.722 (95% 0.663–0.780), and 0.775 (95% 0.717–0.832), respectively.

A multivariate logistic regression analysis revealed that after adjusting for confounders NAR upon admission was associated with POP [odds ratio (OR) = 1.907; 95% confidence interval (CI), 1.232–2.953; *p* = 0.004; Table 2]. Age (OR = 1.068; 95%CI, 1.029–1.108; *p* < 0.001), initial mFisher grade ≥ 3 (OR = 2.512; 95%CI, 1.199–5.264; *p* = 0.015), initial NLR (OR = 1.078; 95%CI, 1.034–1.123; *p* < 0.001), and the rate of mechanical ventilator use (OR = 7.680; 95%CI, 2.518–23.430; *p* < 0.001) remained as independent factors of POP. In the ROC curve analysis used for predicting POP after aSAH, NAR value of 0.27 was determined to be the cut-off value (AUC [95% CI] 0.775 [0.717–0.832]; *p* < 0.001; Youden's index = 0.41; Figure 2), we also computed the AUC of WFNS grade (95% CI, 0.725 [0.654–0.796]; *p* < 0.001; Figure 2) and NLR (95% CI, 0.722 [0.663–0.780]; *p* < 0.001; Figure 2) in order to compare the predictive power between NAR and conventional markers. The results were able to show that the predictive performances of NAR were comparable to WFNS grade (NAR vs. WFNS grade: *Z* = 1.587, *p* = 0.112; Figure 2) and NLR (NAR vs. NLR: *Z* = 1.335, *p* = 0.182; Figure 2).

**Table 2:** Parameters related to the occurrence of postoperative pneumonia using multivariate logistic regression analysis in patients with aneurysmal subarachnoid hemorrhage.

	**Odds ratio**	**95% confidence interval**	***P-*value**
Age	1.068	1.029-1.108	<0.001
mFisher ≥ 3	2.512	1.199-5.264	0.015
NLR	1.078	1.034-1.123	<0.001
NAR	1.907	1.232-2.953	0.004
Mechanical ventilator used	7.680	2.518-23.430	<0.001

Finally, the patients were split into two groups, the high and the low NAR groups, according to the best cut-off value of NAR. Patients in the former NAR group had higher hypertension, higher initial WFNS, higher rates of POP, and a higher rate of mechanical ventilator use than those within the low NAR group (Table 3).

**Table 3:** Demographics and baseline characteristics of aneurysmal subarachnoid hemorrhage patients by the NAR cut-off value.

**Characteristics**	**NAR ≥ 0.27** **(*n* = 151)**	**NAR < 0.27** **(*n* = 244)**	***P-*value**
**Demographics**			
Age(years)	62.0(51.0-67.0)	61.5(52.0-66.0)	0.934
Gender (female)	97(64.2%)	177(72.5%)	0.083
**Prior medical history**			
Hypertension	118(78.1%)	164(67.2%)	0.02
Diabetes	9(6.0%)	15(6.1%)	0.94
**Admission status**			
WFNS grade			<0.001
<Grade III	97(64.2%)	223(91.4%)	
≥Grade III	54(35.8%)	21(8.6%)	
**Admission CT**			
mFisher grade			<0.001
< Grade III	100(66.2%)	218(89.3%)	
≥Grade III	51(33.8%)	26(10.79%)	
IVH	56(37.1%)	43(17.6%)	<0.001
**Aneurysm characteristics**			
Location			0.028
anterior circulation	140(92.7%)	238(97.5%)	
posterior circulation	11(7.3%)	6(2.5%)	
Treatment			0.475
Clip	132(87.4%)	207(84.8%)	
Coil	19(12.6%)	37(15.2%)	
POP	54(35.8%)	24(9.8%)	<0.001
Mechanical ventilator used	42(27.8%)	9(3.7%)	<0.001

## Discussion

To our knowledge, no other studies have explored the association between NAR and POP after aSAH, in this study, we have found that a higher NAR value on admission was related to POP in patients with aSAH. Interestingly, NAR remained a significant factor of POP after adjusting for traditional risk factors such as age, clinical grade, and a higher rate of mechanical ventilator use.

Postoperative pneumonia is a common condition with potentially severe complications within 30 days after surgical treatment of patients with aSAH, directly affecting the prognosis (8, 21, 22). Although there is a variety of classic predictors of POP, most are still based on clinical parameters such as age and WFNS grade (7), whereas the relevant biomarkers that have been used in recent years to predict the occurrence of POP after aSAH include cerebral lactate and NLR (12, 23). We suppose that hematology inspection data might be more productive in showing objective subtle changes in patients, especially when both hematology indicators were administered in combination.

Previous studies have shown that NAR was an inflammatory marker for cancers, sepsis, in-stent restenosis undergoing carotid angioplasty, and STEMI (13–16), but the underlying mechanisms have remained unclear. Other studies have also shown that neutrophil counts increase as part of the post-stroke immunodepression phenomenon (24–26), which is activated by the sympathetic nervous system and the hypothalamic–pituitary–adrenal axis (27, 28). Not only is serum albumin, the most abundant antioxidant in the living body, but it is also an inflammatory biomarker that is involved in systemic inflammation through high levels of pro-inflammatory cytokines and growth factors (29–31).

Moreover, other studies have shown that albumin therapy can enhance organ function and minimize complications in patients with aSAH, which may lead to a better outcome (32–34). We argue that pathological mechanisms of elevated NAR related both to an excessive neuroinflammation response, indicated by increased neutrophil, and albumin decrease that might result in susceptibility to infection as POP. Some of these studies also demonstrated a close relationship between POP and severe aSAH (7, 35).

The results presented in this study showed that NAR levels in patients with aSAH with higher WFNS and mFisher grades were higher than those with lower WFNS and mFisher grades, which conclude that the severity of aSAH could be associated with NAR and POP. Additionally, the incidence of pneumonia in patients with aSAH with higher WFNS grade was higher than those with lower WFNS grade.

The ROC curves were further applied to discriminate between patients with aSAH at risk of POP, having observed that NAR was significantly capable of predicting the occurrence of POP. Although inflammatory conditions may be dynamically changing during the early acute period, these predictive abilities were comparable to the NLR and WFNS grades. Our findings, in turn, suggest that the NAR may be potent enough to predict POP.

At the same time, it is interesting to find a higher level of NAR in the POP group than that found in the non-POP group, regardless of using the ventilator or not. This is probably due to the internal inflammation states remaining an imperative cause of pneumonia in patients, despite the risk of lung infection due to mechanical ventilation, and NAR might ultimately be a promising predictor of POP.

## Study Limitations

The present study has several limitations that ought to be addressed. It consists of a retrospective single-center analysis in which the possibility of a selection bias may exist, making the generalization of these results and their relation to clinical conditions a cautionary matter. Moreover, whether NAR changes over time are correlated with the onset of POP needs to be further examined.

## Conclusion

The current study suggests that NAR might be considered a robust and readily available biomarker whose usefulness aims to predict POP occurrence after aSAH. This study can provide an insight into preventive antibiotic therapy and albumin therapy, which ultimately suggests the usefulness of further clinical trials on this matter.

## Data Availability Statement

The raw data supporting the conclusions of this article will be made available by the authors, without undue reservation.

## Author Contributions

XZ and CW helped with data collection. XZ and AL contributed significantly to the analysis and manuscript preparation. XZ, SZ, RL, and CW performed the data analysis and wrote the manuscript. XZ, SZ, AL, and RL helped perform the analysis with constructive discussions. All authors contributed to the article and approved the submitted version.

## Conflict of Interest

The authors declare that the research was conducted in the absence of any commercial or financial relationships that could be construed as a potential conflict of interest.

## Publisher's Note

All claims expressed in this article are solely those of the authors and do not necessarily represent those of their affiliated organizations, or those of the publisher, the editors and the reviewers. Any product that may be evaluated in this article, or claim that may be made by its manufacturer, is not guaranteed or endorsed by the publisher.
